# Towards human exploration of space: the THESEUS review series on neurophysiology research priorities

**DOI:** 10.1038/npjmgrav.2016.23

**Published:** 2016-08-18

**Authors:** Olivier White, Gilles Clément, Jacques-Olivier Fortrat, Anne Pavy-LeTraon, Jean-Louis Thonnard, Stéphane Blanc, Floris L Wuyts, William H Paloski

**Affiliations:** 1 Cognition, Actions et Plasticité, Sensorimotrice, INSERM UMR1093, UFR STAPS, Université Bourgogne Franche-Comté, Dijon, France; 2 International Space University, Strasbourg, France; 3 Centre Hospitalier Universitaire, Explorations Fonctionnelles Vasculaires, UMR CNRS 6214 Inserm 1083, Angers, France; 4 Centre Hospitalier Universitaire, Département de Neurologie, Toulouse, France; 5 Institute of Neuroscience, Université catholique de Louvain, Brussels, Belgium; 6 Université de Strasbourg, IPHC, Strasbourg, France; 7 CNRS, UMR 7178, Strasbourg, France; 8 AUREA - BIMEF, University of Antwerp, Antwerp, Belgium; 9 University of Houston, Health and Human Performance, Houston, TX, USA

## Abstract

The THESEUS project (Towards Human Exploration of Space: a European Strategy), initiated within the seventh Framework Programme by the European Commission, aimed at providing a cross-cutting, life-science-based roadmap for Europe’s strategy towards human exploration of long space missions, and its relevance to applications on Earth. This topic was investigated by experts in the field, in the framework of the THESEUS project whose aim was to develop an integrated life sciences research roadmap regarding human space exploration. In particular, decades of research have shown that altered gravity impairs neurological responses at large, such as perception, sleep, motor control, and cognitive factors. International experts established a list of key issues that should be addressed in that context and provided several recommendations such as a maximal exploitation of currently available resources on Earth and in space.

## Introduction

Neurological responses to the spaceflight environment challenge the performance of crewmembers at critical times during spaceflight missions (see e.g., ref. 1). Operational performance may be impaired by spatial disorientation,^2–4^ perceptual illusions,^5–7^ balance disorders,^8^ motion sickness,^9,10^ and altered sensorimotor control,^11,12^ all of which are triggered by *g*-transitions and persist for some time after as the neurological systems adapt to the new gravitational (or gravitoinertial) loading. These neurological changes may have adverse effects on crew cognition, spatial orientation, control of vehicles, and other complex systems, and dexterous manipulation skills. Although crewmembers eventually adapt to new gravitational environments (e.g., microgravity), subsequent transitions back to the old environment (e.g., 1*g*) or to a new environment (e.g., 1/6*g* on the Moon or 3/8*g* on Mars) will cause a new disruptions to these systems, impairing performance until readaptation (or new adaptation) has occurred. Following such transitions, crewmembers may be unable to accomplish certain critical entry, landing, and post-flight physical activities. Current methods of pre-flight training and post-flight rehabilitation have not been optimized to minimize the functional impacts of these natural adaptive responses during *g*-transitions or to restore environment-appropriate sensorimotor functions after *g*-transitions.

Although we have sensory organs that specifically detect changes in the accelerations and forces acting on our bodies,^13^ we have no specific gravity receptors. Instead, the direction and strength of the gravity (or gravitoinertial) vector are deduced from central integration of information from many types of sensory receptors distributed throughout our body, including visual, proprioceptive, haptic, and vestibular receptors. This process is termed graviception. Nevertheless, the morphology and physiology of our motor systems have evolved such that the repertoire of movement paradigms enabled our survival by accommodating the physical properties of the environment, including gravity. This has resulted in numerous highly integrated motor control mechanisms that are clearly designed and calibrated to function in a 1-*g* environment. One means of understanding how these control mechanisms function is to investigate how they adapt when the gravitational environment has changed.

Changes in sensorimotor functions resulting from changes in gravitational loading also appear to have collateral effects on muscle atrophy, cardiovascular deconditioning, and cognitive deficits, such as poor concentration, short-term memory loss, and alteration in spatial representations, known to occur during spaceflight.^2,4,14,15^ Changes in sensorimotor functions are probably not the only factors influencing these other flight-associated changes, but their contributions should be recognized and better understood. Therefore, critical questions should be investigated regarding the functioning of biological sensors relevant for the integration of gravity, motor systems, and spatial orientation and cognition in both normal and altered gravity. An integrative approach should be employed to address these questions. Also, the effects of space radiation on the central nervous system need to be better understood, as the potential for damage to neural structures controlling critical life functions and/or adaptive responses is unknown, and methods for detecting and protecting or repairing such damage are poorly understood.

## Organization of this review

The review will present four key issues raised by the expert group as well as by the scientific community. These critical questions contribute to a great uncertainty in the ability to conduct long-duration exploration of near-earth objects and consequently, travel to Moon or Mars. Each of these four points will be detailed hereafter with the same structure. After a specific introduction of the topic, relevance for space missions will be emphasized. Then, research gaps will be highlighted and proposed research investigations will be presented. Many features of the key questions outlined here present cross-disciplinary aspects with other physiological systems. Therefore, the description of each key issue ends with the identification of specific links with other scientific domains. This review concludes by highlighting potential benefits of the recommended research for Earth application and health.

## Key issue 1: impacts of long-duration spaceflight on the senses

Since the first space missions, we know that changes in gravity alter the structure and/or performance of various sensory systems (e.g. vestibular, proprioceptive, tactile, olfactory, baroreceptors, and so on) as well as basic perceptions associated with these systems (e.g. graviception, spatial orientation, self-motion, and so on). However, details and mechanisms of these changes during and after *g*-transitions have not yet been clearly elucidated.

### Relevance for space exploration missions

Altered sensory or perceptual functions can lead to performance decrements,^14,16^ spatial disorientation, or altered situational awareness during critical vehicle control operations that may seriously endanger the mission and the crew.^1^ The causes and effects of reported in-flight changes of visual acuity on visual perception and performance remain an open question.^17^ The effects of Extra Vehicular Activities (EVA) on sensory and perceptual functions have barely been studied, for instance for EVA height vertigo.^18,19^ Furthermore, spaceflight-induced changes in sensory and perceptual systems may alter interactions with other physiological systems (muscle, cardiovascular, and so on), further contributing to reduced performance capabilities of the individual.

### Knowledge gaps

While the effects of sustained 0*g* on some sensory and perceptual functions have been well studied,^12,20–22^ little is known about the effects of sustained hypo-*g* (0<*g*<1) on these functions. These ranges of gravitational forces have only been explored since recent parabolic flight experiments mimicking Martian (0.38*g*) or Lunar (0.16*g*) gravities. Similarly, while the transient (adaptive) effects on some sensory and perceptual functions have been well studied following shifts from 0 to 1*g* (and, to a lesser extent, 1 to 0*g*), little is known about the transient effects on these functions following shifts from 1*g* to hypo-*g* (and vice versa).

Most investigations that addressed adaptation in new gravitational environments focused on distinct stable phases, such as the 0, 1 and 1.8*g* in parabolic flights. However, gravity may also vary significantly and continuously over time, e.g. during launch and entry phases of a spacecraft. When confronted with a radically new environment, humans need time to reestablish causality between actions and their consequences. This already takes time in a stable new dynamical context. However, it poses even more challenges when the underlying environment is also varying, bringing an additional cloud of uncertainty over motor processes. In that context, little is known about what happens to sensory and perceptual functions during *g*-transitions, where gravitoinertial forces can vary by 2–3*g* or more during periods of 10–30 min, from any starting *g*-level to any finishing *g*-level.

Space missions, for a given astronaut, are always separated by long interval of times (months to years), allowing the various systems to reset. The responses of the central nervous system to repeated *g*-transitions associated with repeated space missions have shown some tendencies toward dual-adaptation (e.g., response severities decrease as the number of missions increases). However, responses to significant *g*-level changes with shorter recovery times such as during intermittent artificial gravity exposures (every few hours to days) are largely unknown (but refer to ref. 23 for a review of context-specific adaptation). How much time is required for significant adaptive responses to manifest themselves during/after *g-*transitions, and what effects are driven by subsequent *g*-transitions during/after this time period?

Sustained hypo-*g* fields cannot be created on Earth, but sustained hyper-*g* (*g*>1) fields can be generated easily using rotating habitats. Artificial gravity created by rotating all or part of a space vehicle might prove to be an efficient, effective multi-system countermeasure. However, the unusual force environment created by the rotation (i.e., *g*-gradients, Coriolis forces, and cross-coupled angular accelerations) will likely affect sensory and perceptual functions. The magnitudes, time courses, reversibility, and consequences of these effects are largely unknown although many robotic experiments focused on adaptation after focal perturbation of e.g. the upper limb.^24^ Interestingly, should we take into account the above-mentioned side effects of centrifugating habitats,^25^ could transitions between hyper-*g* and 1*g* be used as a valid experimental model for examining neurophysiological responses to transitions between 1*g* and hypo-*g*?

From a more neurological aspect, the effects of pharmaceutical countermeasures (e.g., promethazine) on sensory and perceptual functions have not been studied in any non-terrestrial environment. This point should not be neglected because most participants of parabolic flights take medication. Furthermore, does gravity alter the structure of neurological systems such as end organs, the cerebellum, the brain stem, the cortex, or the spinal cord? A recent brain-imaging report demonstrated differences in resting-state functional connectivity between motor cortex and cerebellum, as well as changes within the default mode network.^26^ Further, are there any long-term or irreversible health or performance consequences associated with any such structural changes? Finally, apart from gravity itself, space travelers will be exposed to radiation. What are the potential effects of these ionizing radiations on sensory and perceptual functions? Are they predictable? What countermeasures can be used to detect and/or minimize these effects? What rehabilitation protocols can be used to aid recovery from these effects?

### Recommendations for future research

Europe is well-equipped to address most if not all the questions raised above. First, on a theoretical basis, there is a need to quantify the relationships between *g*-level and sensations/perceptions. In other words, what are the *g*-thresholds above which the various sensations and perceptions are affected? Adaptive responses between two different environments can follow different time scales. For instance, are time courses associated with adaptive responses of the sensory and perceptual systems between 1*g*/0*g* and 0*g*/hypo-*g* different? Moreover, studies should also focus on adaptation mechanisms of the sensory and perceptual systems in rapidly changing gravitational environments.

When we plan any action within the environment, we need some fixed reference. When gravity is altered, our central nervous system lacks that reference that defines verticality and calibrates our senses. In that case, a new reference needs to be inferred through the integration of our biological senses, such as audition, haptics, proprioception, smell, vision, taste, and so on. The effect of gravity on each specific sense should be studied.

**Figure 1:**
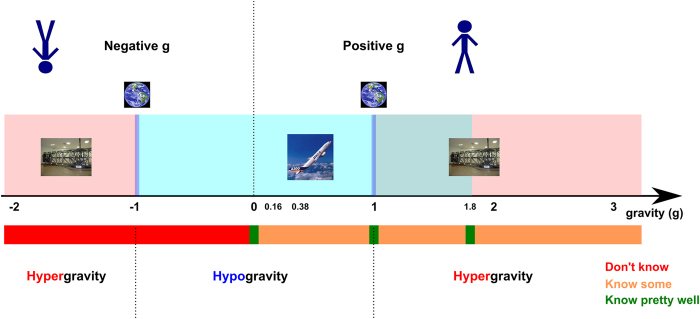
Gravity spectrum currently explored in motor control and human neurophysiology. Gravity is represented on the horizontal axis. Centrifuges can be used to explore the red zone above the axis and parabolic flights can be used to explore the blue zone. The special conditions at +1 g and −1 g can be tested on the ground. Interestingly, the interval 1–1.8*g* can be studied both in centrifuges and parabolic flights. Below the axis, the rectangle quantifies the knowledge we have about motor control across gravitational environment (see key for the rectangles).

Europe and the International community do not need further fundamental methodological developments to explore the complete spectrum of gravity states (Figure 1). On one hand, hypergravity—either below −1*g* or above +1*g*—can be simulated with nearly arbitrary profiles in a human centrifuge. Time is also much less constrained in those experiments than in the ISS. In addition, parabolic flights can be used to explore hypogravity between 0 and ca 2*g*, with, however, more constraints on gravitational profiles and times. More fundamentally, negative hypogravity could easily be explored as well, but ethical considerations must be considered because it would involve flipping the participant upside down. One can even exploit redundancy. Centrifuge-based experiments and parabolic flights could both be used to explore the 1–2*g* overlapping interval providing a mean to disentangle between potential confounding factors such as stress. Furthermore, short-radius centrifuges also offers the possibility to investigate how the central nervous system adapts to a position-dependent gravitoinertial vector. Many of the open questions outlined above can be addressed with these methods, both with naive inexperienced participants and with subjects who have accumulated significant altered gravity experience (i.e., many parabolas or astronauts). Pharmaceutical countermeasures (e.g., promethazine) on sensory and perceptual systems should be studied in these space analogs. Finally, the use of commercial suborbital flights should be considered as a suitable platform for the study of transient responses to changing *g*-levels.

Laboratory experiments are always focused on specific questions, therefore simplifying the context. The drawback of this approach is that we hardly ever test ecologically valid movements. Consequently, we could take the opportunity to evaluate the effects of EVAs on sensory and perceptual functions by performing experiments during scheduled EVAs. This should provide a window into a comprehensive strategy adopted by astronauts, optimizing their resources.

Basic effects of gravity and radiation on biological tissues should also be further tested. Ground-based accelerator exposure facilities provide beams of protons and HZE particles, at energies within the range of space radiation. These means are suitable to investigate the consequences of ionizing radiation on neurological functions. Structural changes in the nervous system of animals can be investigated in small, animal centrifuges.

### Transdisciplinary aspects

As already mentioned several times, strong links have to be emphasized with issues related to radiation in space. The longer a crew member is exposed to the radiation environment of space, the more likely it is that some high-energy particle will cause significant damage to a critical central nervous system neuron/structure that could lead to permanent, functionally relevant structural changes. What are the potential effects of radiation on neurologic functions? What countermeasures can be used to detect and/or minimize these effects?

## Key issue 2: impacts of long-duration spaceflight on sensorimotor performance

Changes in gravity have been shown to alter sensorimotor control in general, and affects goal-directed eye, hand, head, limb, or body movements,^27–31^ eye–head coordination,^27^ eye–hand coordination,^32^ dexterous manipulation,^33–37^ postural control,^1,10,38–40^ locomotor control,^10,41^ and tilt perception.^42^ However, details and mechanisms of these changes during and after *g-*transitions have not yet been clearly elucidated, nor have their functional/operational significance.

### Relevance for space exploration missions

Altered sensorimotor control performance during critical mission activities may also seriously endanger mission objectives, mission equipment, or the crew. Furthermore, altered sensorimotor control performance during typical activities of daily living after return from space missions may seriously endanger crew. These activities include: controlling space vehicles (e.g., launch, entry, landing, rendezvous, docking, and so on), ground-based vehicles (e.g., rovers, automobiles, watercraft, aircraft, and so on), or remote manipulators (e.g., robotic arms), performing Extra Vehicular Activities, emergency egress, or exercise/sport/athletic activities, and so on.

### Knowledge gaps

The understanding of adaptation necessitates access to baseline astronaut performance as well as a snapshot measurement of their behavior immediately upon return. Most of the time, these data are only available at least 24 h after landing, which is too long a delay to catch effects that fade out quickly. More recently, the current NASA/Russia Field Test is acquiring the data within hours of return from ISS. Furthermore, the inaccessibility of operational performance data by the scientific community complicates the study of the relationships between physiological changes and operational performance decrements (e.g., piloting performance, emergency egress, manual control, and so on).

The mechanical control of eye movements is not very sensitive to gravity because the eyeball has a small inertia compared with, e.g, the upper limb. For that reason, gaze strategies can be utilized as a window into the working of how we coordinate movements.^32,33^ However, in order to fully benefit this opportunity, we still lack the understanding of natural three-dimensional eye movements, particularly during spaceflight (e.g., the targets for smooth pursuit and saccade movements have only been tested using a fronto-parallel screen). Control of vergence movements, in particular, are poorly understood. More comprehensively, few mathematical models exist to describe sensory motor adaptive responses, especially to altered gravity, and no model incorporates eye movements.

In terms of space analogs, what ranges of radii and angular velocities are required for continuous artificial gravity (e.g., rotating vehicle) to appropriately stimulate the load/acceleration receptors while minimizing the Coriolis forces and cross-coupled angular velocity side effects? We also lack quantitative information about how intermittent artificial gravity might benefit sensorimotor systems, as well as the heart, bone, muscle, and cardiovascular systems. Are supplemental (concurrent) exercise countermeasures required to optimize this putative countermeasure?

### Recommendations for future research

Protocols should be established to disclose the operational performance data to qualified researchers. Developing closer partnerships among the scientific, operations, and training communities might be the best way to achieve this in close relation with ethical boards.

As most of the issues associated with altered sensory or perceptual functions could lead to decreased sensorimotor performance, all of the recommendations under Issue 1 also apply to this Issue.

Measurements of three-dimensional eye movement control performance during spaceflight and EVA in particular should be envisaged. The development of new mathematical models should include an explicit dependency on gravity to simulate sensorimotor responses to changed gravity.

Research should be carried out in space analogs and in-flight artificial gravity experiments to determine the optimal radii and angular velocities to promote multi-system (including vestibular-sensorimotor) protection from spaceflight adaptation issues. A long-term goal would be to set up a flight-simulator in space (centrifuge based, if possible) to correlate landing performance with in-flight training.

### Transdisciplinary aspects

Overarching aspects linking to all scientific disciplines are clearly evident. The inaccessibility of operational performance data to the scientific community complicates the study of the relationships between physiological changes and performance decrements (e.g., piloting performance, emergency egress, manual control, and so on) during critical mission phases. Protocols should be established for disclosure of operational performance data to qualified researchers. Developing closer partnerships among the scientific, operations, and training communities might be the best way to achieve this. Furthermore, the setting up of a shared database recording past experimental data sets and information on their contexts could be very valuable to enhance the usability of the previous data (e.g., challenge the low-*N* problem in space research).

## Key issue 3: impacts of neurophysiological changes on spaceflight-induced decrements in neurobehavioral performance

Changes in gravity have been observed to decrease work capacity, vigilance, cognition, motivation, and other aspects of neurobehavioral performance.^16^ However, details and mechanisms of these changes, especially the role that altered senses and perceptions might play during and after *g-*transitions, have not yet been clearly elucidated.

### Relevance for space exploration missions

The consequences of altered work capacity, vigilance, cognition, and motivation during spaceflight could range from decreased crew efficiency to loss of mission and crew. Current countermeasures against motion sickness during and after flight present unacceptable risks to crewmembers^43,44^ and may be inappropriate for exploration missions.

### Knowledge gaps

The incidence and severity of mission-related operational performance decrements are unknown, except in a few publicly observed examples. The inaccessibility of operational performance data by the scientific community complicates the study of the relationships between neurophysiological changes and neurobehavioral decrements. Mechanisms of decreased or off-nominal performance during mission critical operational tasks (e.g., vehicle control) are unknown. Although links between vestibular dysfunction and cognitive deficits have been established on Earth,^45^ no such links have been investigated in space research. The effects of ground-based and in-flight training regimens on neurobehavioral decrements have not been well studied. Finally, effective motion sickness countermeasures having minimal effects on neurobehavioral or sensorimotor function are currently unknown. Ease of administration of medication against space motion sickness, such as intranasal, should also be considered.

### Recommendations for future research

Studies should be performed to elucidate the time course and severity of spaceflight-induced cognitive deficits (e.g., Sopite syndrome (drowsiness), space fog, also known as ‘mental viscosity’ or ‘space stupids’), focusing on the role of neurophysiological changes. The effects of ground-based and in-flight training regimens on neurobehavioral decrements should be further investigated. Effective motion sickness countermeasures having minimal effects on neurobehavioral or sensorimotor function should be sought.

### Transdisciplinary aspects

Links are clearly evident with psychology and human–machine systems as well as with health care aspects. Besides, five transdisciplinary aspects need to be highlighted:

### Cognitive function

Cognitive function comprises several tasks such as attending, selecting, decision-making, recognizing, imitating, and remembering that involve different parts of the brain. To fully assess the effects of spaceflight on cognitive function, and to better characterize the transient spaceflight phenomenon sometimes referred to as the ‘space stupids,’ each of these cognitive function tasks needs to be assessed individually with specific tests. More particularly, we should further investigate how changes in gravitational stimulation of vestibular receptors affect cognitive function and what training methods can be used to reduce these effects. In case of pharmacological countermeasures against motion sickness, how do medications used to relieve these symptoms affect cognitive function? What alternative medications could be used to reduce these effects?

### Motivation and vigilance

Similarly, how do changes in gravitational stimulation of vestibular receptors and/or symptoms of motion sickness affect crew motivation, sleep, and vigilance? What training methods or medications could be used to reduce these effects, without compromising critical psychological abilities?

### Sleep and circadian rhythms

There is a growing body of evidence suggesting that chronic sleep loss, which has been reported to occur commonly during spaceflight,^46^ is associated with neurodegenerative, endocrinological, immunologic, affective, and cognitive/memory, and motor performance deficits.^47–49^ The mechanisms of in-flight sleep loss are not well understood. Many factors may have a role, including light conditions, psychological issues, and vestibular stimulation by gravitoinertial forces.^50–52^ What is the relative importance of these factors? How do changes in gravitational stimulation of vestibular receptors affect circadian rhythm? What training methods or medications could be used to minimize these effects?

### Spatial, geographic, and situational awareness

We know from decades of research that the omnipresence of gravity provides a strong reliable calibration signal for our various sensory organs.^2,53,54^ How does loss of the fundamental spatial orientation reference provided by gravity affect spatial processing? What affects does this have on spatial, geographic, and/or situational awareness? What training methods or physical aids could be used to minimize these effects?

### Performance

Previous work has suggested that similar neural substrates are involved in movement execution, observation, and motor imagery,^55,56^ and that the same descriptive laws of movement control apply to these processes.^57^ How could motor imagery and/or observation be used to aid crewmembers in efficiently learning and achieving specific physical tasks in altered gravity?

## Key issue 4: countermeasure strategies to minimize the risks associated with neurophysiological changes during and after *g*-transitions

### Relevance for space exploration missions

Crewmembers are called upon to perform many of the riskiest tasks associated with spaceflight mission during and after *g-*transitions, which are also the times of greatest environmental challenges to the neurological systems during a mission. As noted above in Issues 1–3, altered sensations and perceptions, sensorimotor performance, or cognition during these critical mission phases could seriously endanger the mission and the crew.

### Knowledge gaps

First and again, the inaccessibility of the operational performance data to the scientific community complicates the study of the relationships between physiological changes and performance decrements (e.g., piloting performance, emergency egress, manual control, and so on) during these critical mission phases. Second, only limited research data have been collected during *g*-transitions to and from spaceflight. Third, the effects of pre-flight training or other countermeasures on performance during these phases have not been evaluated.

### Recommendations for future research

Studies of crew neurophysiological responses and crew performance should be conducted during and immediately after launch/insertion and entry/landing. The incompressible 24-h delay is too long to allow accurate measurements of effects. Ideally, this precious information should be included together with their context in a shared database. Further, the effectiveness of operational training protocols and recency, the propensity to recall the last memorized events,^15^ as well as pre-flight approaches designed specifically for these phases should be studied.

### Transdisciplinary aspects

In addition, two important issues should be highlighted:

Loss of motor tone: How do changes in gravitational stimulation of vestibular and/or peripheral load receptors affect tonic spinal–motor activation of anti-gravity muscles? What role does this play in muscle atrophy and/or (indirectly) bone loss? What role does it play in post-flight orthostatic function? What countermeasures should be used to minimize these effects?

Vestibular control of cardiovascular regulation: What role do vestibular afferents play in the autonomic regulation of myocardial contractility, vascular tone, plasma volume, and other aspects of cardiovascular regulation? How do changes in gravitational stimulation of vestibular receptors affect cardiovascular regulation? As the number of mixed gender crew increases, the importance of studies designed to highlight how orthostatic differences in male and female alter motor functions also grows.^58^ What countermeasures should be used to minimize these effects?

### Conclusion and earth benefits

The human nervous system has evolved to respond to gravitational conditions.^59–64^ On Earth, the presence of gravity-mediated inputs from an ensemble of somatic receptors sensitive to force and acceleration generates a gravitational reference frame from which spatial orientation can be deduced and movements can be planned and executed.^53^ Changes in this fundamental reference signal caused by spaceflight or other sustained gravitoinertial force fields cause transient disruptions in performance until the central integration functions can adapt to the altered reference stimuli.^14,21,28–31,65,66^ Crew health, safety, performance, and, eventually, mission success, can only be assured after effective countermeasures are developed to optimize the adaptation to new gravitoinertial environments. But, such countermeasures cannot be developed without understanding the fundamental mechanisms underlying the adaptive processes involved. Many ground-based studies address learning and transfer of motor skills into novel environments. However, only spaceflight studies can examine the nervous system responses to reduced gravity. Thus, we conclude that a series of basic and operational scientific experiments into the neurophysiological responses to spaceflight are required to enable the next steps in human exploration of space.

Many proposed research investigations are also relevant to shed light on issues encountered on Earth. Spatial disorientation and situational awareness issues cause up to a quarter of civil aviation accidents. Diminished manual flying skills during visual flight rules piloting is an increasing problem, especially for search and rescue helicopter pilots required to fly with diminished visual cues. Physical aids (e.g., tactile situational awareness system) and countermeasures developed to aid space travelers might also be useful for commercial and military aviation. As graviception has a critical role in spatial orientation perception as well as control of balance, locomotion, and dextrous manipulation, space neurophysiology research will help us to better define the mechanisms underlying the fundamental role of gravity in motor control. It should also help us to better understand the interplay between nervous system function and functioning of the cardiovascular^67^ and muscular systems as well as some of the cognitive aspects of behavioral performance.

The altered gravity environments available during spaceflight offer platforms to study the basic neurophysiology of dexterous manipulation (eye–hand coordination), balance and locomotion, and vehicle control, providing knowledge that serves to help patients with vestibular, neurological, and motor control problems as well as the elderly, which will become a major issue in coming decades. Knowledge gained from studying the training and rehabilitation protocols developed for use with astronauts can be transferred almost directly to patients with specific lesions or disorders requiring retraining or rehabilitation. Finally, mathematical models that have an explicit dependency on gravity could simulate and help make predictions on mechanisms that cannot be observed on Earth. Aged people share coming features with astronauts: some aging-like alterations are observed during spaceflights on a squeezed time scale.^68^ Therefore, cures for what happens in space might be applicable to people with low levels of activity on Earth.

New information from spaceflight studies on the fundamental mechanisms of spatial orientation perception of slopes, depths, and heights may aid architects in optimizing the design of habitats and affordances for the elderly or for those with neurological deficits.

Space provides a unique platform to observe consecutive generations of animals (and eventually humans), born and developed without gravity, allowing the fundamental study of the influence of gravity on development. What is the influence of gravity (or its absence) on development? For example, is there a critical period for development of anti-gravitational reflexes, similar to the critical period for development of vision? Are there synaptic or structural changes in an organism after being in space for long periods of time?
